# The Anti-Inflammatory Effects of Matrix Metalloproteinase-3 on Irreversible Pulpitis of Mature Erupted Teeth

**DOI:** 10.1371/journal.pone.0052523

**Published:** 2012-12-20

**Authors:** Hisanori Eba, Yusuke Murasawa, Koichiro Iohara, Zenzo Isogai, Hiroshi Nakamura, Hiroyuki Nakamura, Misako Nakashima

**Affiliations:** 1 Department of Dental Regenerative Medicine, Center of Advanced Medicine for Dental and Oral Diseases, National Center for Geriatrics and Gerontology, Obu, Aichi, Japan; 2 Department of Advanced Medicine, National Center for Geriatrics and Gerontology, Obu, Aichi, Japan; 3 Department of Endodontics, School of Dentistry, Aichi-gakuin University, Nagoya, Aichi, Japan; University of Bergen, Norway

## Abstract

Matrix metalloproteinases (MMPs) are involved in extracellular matrix degradation and the modulation of cell behavior. These proteinases have also been implicated in tissue repair and regeneration. Our previous studies have demonstrated that MMP-3 elicits stimulatory effects on the proliferation and the migration of endothelial cells as well as anti-apoptotic effects on these cells *in vitro*. In addition, we found that MMP-3 enhanced the regeneration of lost pulp tissue in a rat incisor pulp injury model. However, continuously erupting rodent incisors exhibit significantly different pulp organization compared with mature erupted teeth. Therefore, we have further extended these studies using a canine irreversible pulpitis model to investigate the effects of MMP-3. In this study, the crowns of the canine mature premolars were removed and the pulp tissues were amputated. The amputated pulp tissues remained exposed for 24 or 72 hours to induce mild or severe irreversible pulpitis, respectively, followed by sealing of the cavities. In both models, the whole pulp tissues became necrotic by day 14. In this mild pulpitis model, the regeneration of pulp tissue with vasculature and nerves was observed until 14 days after sealing with MMP-3, followed by extracellular matrix formation in the regenerated pulp tissues until day 28. The treatment with MMP-3 resulted in a decrease in the number of macrophage and antigen-presenting cells and a significant inhibition of IL-6 expression on day 3. The inhibition of MMP-3 activity abolished these anti-inflammatory effects. Immunofluorescence staining demonstrated that MMP-3 was involved in the modification of serum-derived hyaluronan-associated proteins and hyaluronan (SHAP-HA) complexes possibly through the degradation of versican. These results demonstrate that MMP-3 can act as an anti-inflammatory agent and suggest that MMP-3 might represent a useful therapy for the treatment of mild irreversible pulpitis.

## Introduction

Inflammation of the dental pulp is caused by dental caries, which result from bacterial infection. Odontoblasts are the first cells to encounter bacterial antigens in dental caries, after which they rapidly induce the chemotaxis of immature dendritic cells (DCs) [1], [2]. Persistent infection leads to the activation of adaptive immune responses. Pulpal pathosis can take one of the following three forms: reversible pulpitis, irreversible pulpitis and pulp necrosis [3]. If pulp exposure injuries are not treated for infection and re-sealed, the introduction of bacteria into the pulp tissues can readily cause irreversible pulpitis that does not allow for their spontaneous healing, ultimately resulting in the necrosis and death of the pulp tissue [4]. The presence of infections, regardless of how minute, can promote pulpitis/necrosis. Therefore, the entire pulp of a tooth affected by irreversible pulpitis should be removed, regardless of the amount of remaining normal pulp tissue. However, long-term studies have shown that the rate of tooth loss is higher for endodontically treated teeth compared to non-treated teeth, due to fracture, secondary caries and complex restoration-associated problems [5], [6], [7], [8]. This has been one of the central challenges in dentistry, and thus the improvement of antimicrobial agents and restorative materials and the diagnosis of pulp vitality to facilitate the control of infection are great interest [9]. The treatment of irreversible pulpitis aims to restore the original architecture and biological function of the infected pulp tissue by facilitating repair or by allowing for tissue regeneration. This restoration requires the recruitment of stem/progenitor cells and the differentiation of these cells into tissue-specific somatic cells as the result of intrinsic factors and extrinsic microenvironmental cues.

Matrix metalloproteinases (MMPs) are a group of proteolytic enzymes that can degrade the principal components of the extracellular matrix. On the basis of these degradation activities, MMPs are widely believed to play a central role in tissue degradation. Several experimental and clinical data concerning MMPs in the contexts of arthritis and cancer have been reported [10], [11]. A number of MMP inhibitors have exhibited efficacy in animal models of disease and have been used in clinical trials for the treatment of cancer, with some studies focused on rheumatoid arthritis and osteoarthritis. However, the application of MMP inhibitors failed to exhibit significant therapeutic efficacy in any of these human clinical trials [12]. In some instances, these inhibitors resulted in adverse effects including musculoskeletal pain, tendonitis and mild anemia with elevated levels of liver enzymes. These results have necessitated the redefinition of MMP functions. Recent studies have shown that MMPs influence many basic processes, such as cell proliferation, differentiation, angiogenesis and apoptosis [10]. Notably, MMP family proteins elicit dual roles in the pathogenesis of inflammation, stimulating protective innate and/or adaptive immune functions, as well as tissue destruction [13]. In addition, the immune-inflammatory responses and tissue destruction induced by pulpal infection, which decrease the potential for tissue regeneration, should be prevented.

Tissue repair and regeneration is a dynamic and highly regulated process involving cellular, humoral and molecular mechanisms. Some individual components are crucial, and the sum of many healing insufficiencies can lead to chronic or non-healing wounds. A hyaluronan (HA)-rich matrix plays a dynamic role in tissue repair and regeneration by occupying space and recruiting inflammatory cells. The functional versatility of HA may be attributed to its unique physiochemical and biological properties, which are largely dependent on its concentration, its chain length, and the large number of HA-binding proteins (HABPs) that interact with it. Of the identified HABPs, serum-derived HA-associated protein (SHAP) is the only protein that is covalently and tightly bound to HA. The formation of the SHAP-HA complex is associated with many inflammatory diseases; for example, large amounts of SHAP-HA are found in the synovial fluid and hyperplasic synovium of patients with rheumatoid arthritis [14]. Thus, modifications in the HA-rich matrix may affect immune-inflammatory responses.

We previously demonstrated that MMP-3 enhanced the proliferation, migration, and survival of human umbilical vein endothelial cells *in vitro* [15]. Furthermore, the topical application of MMP-3 protein to the injured pulp tissues of rat incisors accelerated tissue regeneration, angiogenesis and reparative dentin formation at significantly higher rates than those observed with the control treatment [15]. However, rat incisors are continuously erupting teeth that exhibit pulp organization that is significantly different than that of mature erupted teeth [16]. Therefore, the injury responses of rat incisors might be quite specialized and unique to continuously erupting teeth. Thus, in this study, we have extended our investigation to a canine model of irreversible mild and severe pulpitis to determine the role of MMPs in inflammation and the resulting damage in the dental pulp tissues of mature erupted teeth.

## Results

### The Establishment of Mild and Severe Irreversible Pulpitis Models

**Figure 1:**
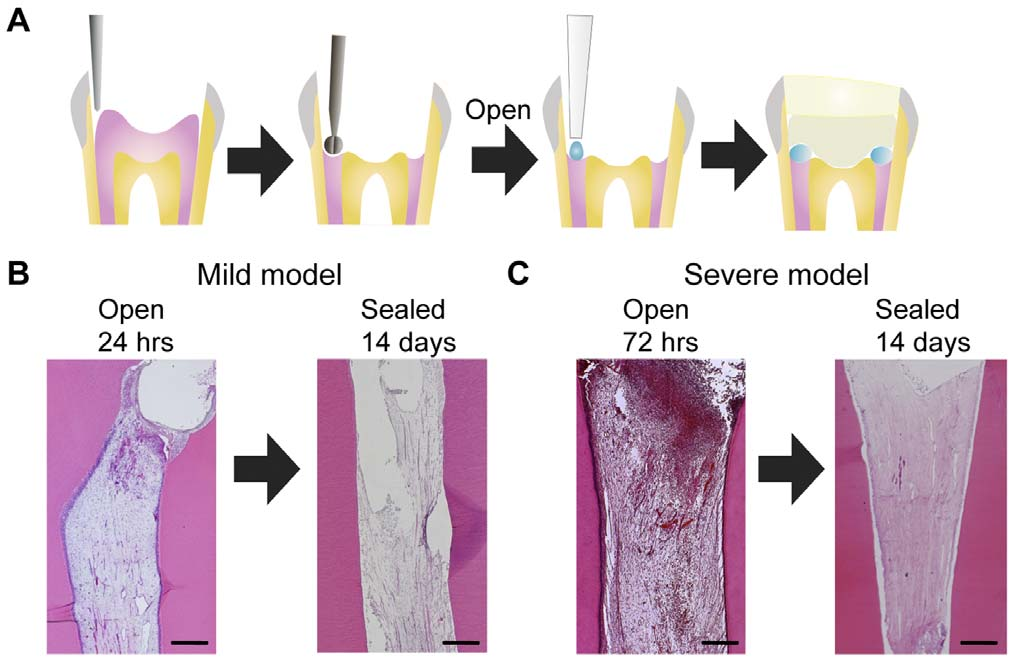
The establishment of mild and severe irreversible pulpitis models. A. Schematic diagrams of the amputation of the dog mature molar pulp tissues and subsequent cavity sealing. The crowns of the upper and lower premolars were removed, and the pulp tissues were amputated using a round burr. The amputated pulp tissues were exposed to allow for infection and treated with solution absorbed in Spongel. After the treatments, the cavity were sealed with phosphate cement and light-cured composite resin. B. The histology of the pulp tissues from dogs with mild irreversible pulpitis. The left panel shows amputated pulp tissue that remained exposed for 24 hours. After 24 hours, the cavity was covered with spongel and sealed. The right panel shows the pulp at 14 days after sealing. C. The histology of the pulp tissues from dogs with severe irreversible pulpitis. The left panel shows amputated pulp tissue that remained exposed for 72 hours. After 72 hours, the cavity was sealed. The right panel shows the pulp tissue at 14 days after sealing. Scale bar, 500 µm.

We performed histological analyses of canine pulp tissues on irreversible pulpitis models established in this study. When the amputated pulp remained exposed for 24 hours, hyperemia, blood vessel dilation and neutrophil infiltration were observed in the upper limited portion of the pulp tissue underneath the amputated site (Figure 1B left panel). When the injured pulp remained exposed for 72 hours, the inflammatory cells were dispersed throughout the upper half of the entire pulp tissue (Figure 1C left panel). The entire pulp tissue was necrotic by day 14 in both of the teeth that were sealed after exposure for 24 or 72 hours (Figure 1B, C right panel), indicating that these teeth could be used as models of irreversible pulpitis. Teeth that were exposed for 24 hours represented a model for mild pulpitis, whereas those exposed for 72 hours represented a model for severe pulpitis.

### Pulp Tissue Regeneration Induced by MMP-3 in the Mild Pulpitis Model

**Figure 2:**
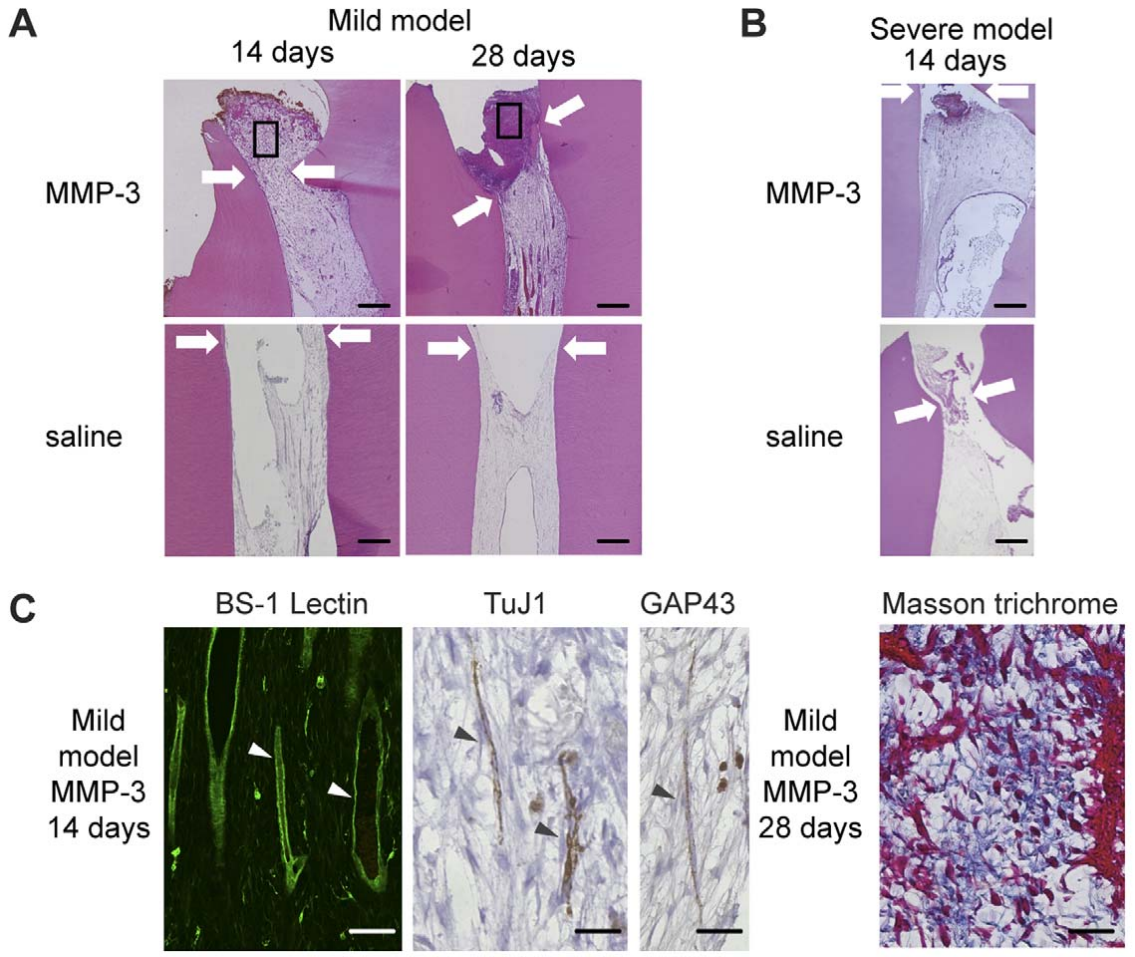
The pulp tissue regeneration induced by MMP-3 in the irreversible pulpitis model. A. H&E staining of pulp tissues from dogs with mild pulpitis at 14 and 28 days after MMP-3 or saline treatment, as indicated. The arrows show the imaginary amputated site, with the indicated areas magnified in C. Scale bar, 200 µm. B. H&E staining of pulp tissues from dogs with severe pulpitis at 14 days after MMP-3 or saline treatment, as indicated. The arrows indicate the imaginary amputated site. Scale bar, 200 µm. C. Immunohistochemical analysis for BS-1-lectin, TuJ1, and GAP43 and Masson’s trichrome staining of pulp tissues from dogs with mild pulpitis at 14 or 28 days after MMP-3 treatment, as indicated. The representative staining of the cells is indicated by arrowheads. Scale bar, 50 µm.

We next investigated the potential therapeutic effects of MMP-3 on the mild and severe pulpitis models. At 14 and 28 days after the sealing of teeth that were treated with MMP-3 in the mild pulpitis model, the pulp tissues were regenerated over the amputated site (Figure 2A upper panel). The pulp tissues of teeth that were treated and sealed with saline in the mild pulpitis model, used as a control, were necrotic at 14 and 28 days (Figure 2A lower panel). In contrast, in the severe pulpitis model, the entire pulp tissues were necrotic at 14 days after both MMP-3 and saline treatments (Figure 2B). Next, we further analyzed these regenerated pulp tissue induced by MMP-3 on the mild pulpitis model. Immunohistochemical analyses using BS-1 lectin staining demonstrated the presence of newly formed blood vessels in the MMP-3-treated pulp tissues on day 14 (Figure 2C). Cells that stained positive for TuJ1 (neuronal marker) and GAP43 (synaptic marker) were also detected (Figure 2C), indicating innervation of the regenerated tissues. Intensely blue-stained collagen in Masson trichrome-stained tissues was observed in the regenerated pulp tissues over the amputated site on day 28 (Figure 2C). In the absence of MMP-3 treatment, immunoreactivity for the same markers was not detected in the necrotic tissues in the mild pulpitis model (data not shown).

### The Suppression of Inflammatory Cell Infiltration by MMP-3

**Figure 3:**
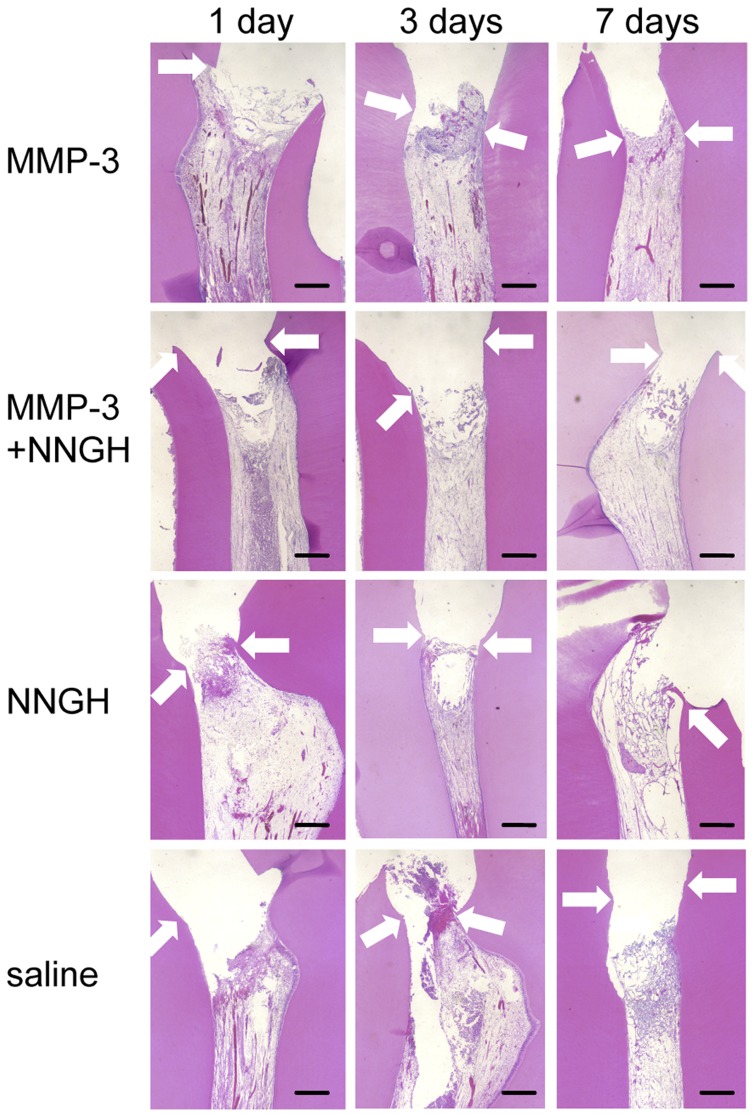
Time course of the histological changes of the pulp tissues from dogs with mild pulpitis. H&E staining of pulp tissues from the dog with mild pulpitis at the indicated number of days after treatment with MMP-3, MMP-3 plus NNGH, NNGH alone or saline alone, as indicated. The arrows indicate the imaginary amputated site. Scale bar, 200 µm.

The mild pulpitis model was assessed over the time course of wound healing and pulp tissue regeneration following MMP-3 treatment. Numerous leukocytes infiltrated into the pulp tissues beneath the amputated site at 1 day after MMP-3 treatment (Figure 3). At 3 and 7 days after MMP-3 treatment, the inflammatory cells disappeared, and the pulp tissues were regenerated over the amputated site. In contrast, a significant number of infiltrated inflammatory cells were also detected in the pulp tissues at 1 and 3 days after treatment with MMP-3 in the presence of a MMP-3-specific inhibitor (NNGH, N-isobutyl-N-(4-methoxyphenylsulfonyl)-glycylhydroxamic acid), NNGH or saline treatment (Figure 3 middle and bottom panel). After 7 days, the entire pulp tissue became necrotic in the MMP-3 and NNGH co-treated group, NNGH-treated group and in the saline-treated group.

**Figure 4:**
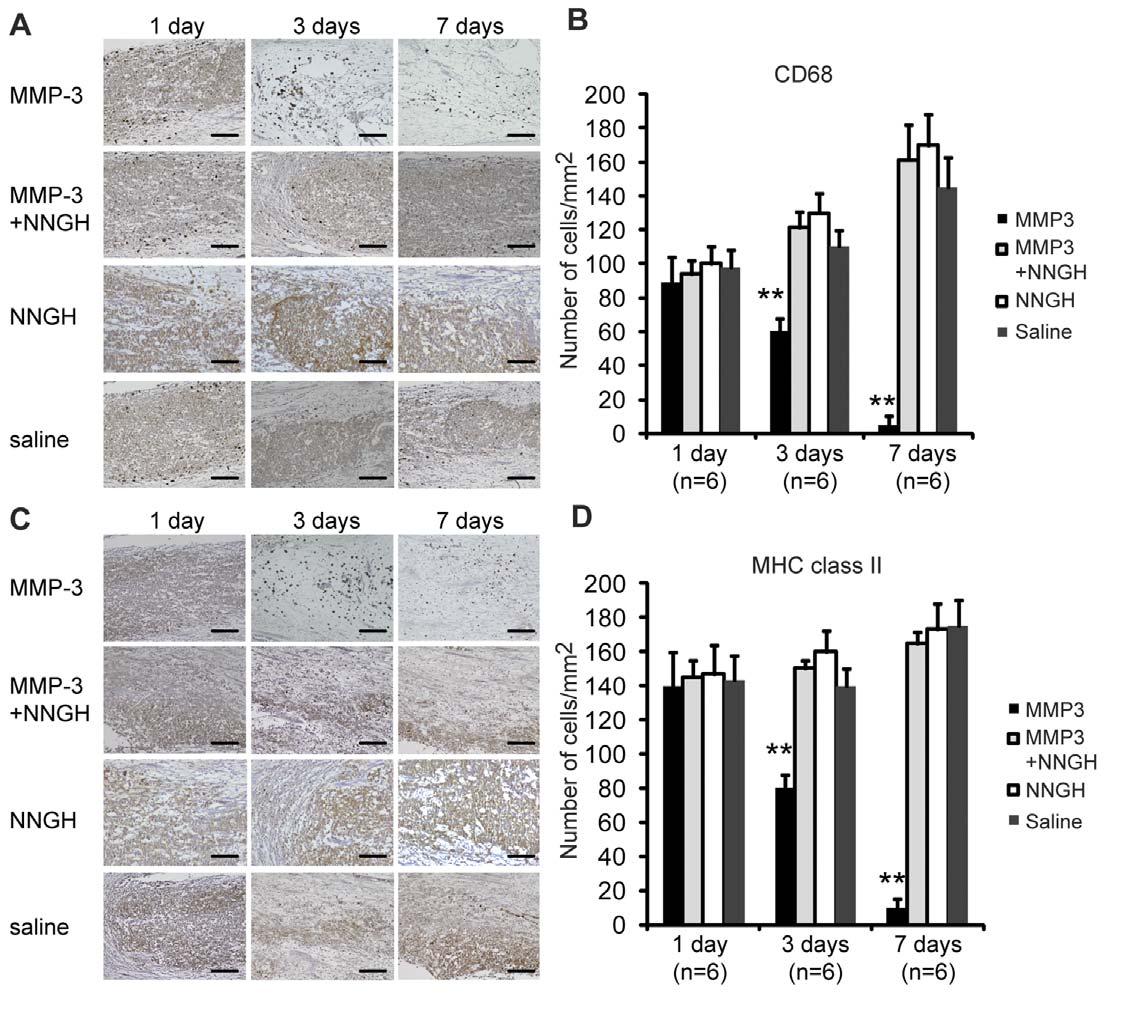
Immunohistochemical analysis of CD68 and MHC class II in samples from dogs with mild pulpitis. A. Immunostaining of pulp tissues using anti-CD68 IgG on the indicated days after treatment with MMP-3, MMP-3 plus NNGH, NNGH alone or saline alone, as indicated. Scale bar, 100 µm. B. Quantitative analysis of the CD68-positive cells on the indicated days after treatment with MMP-3, MMP-3 plus NNGH, NNGH alone or saline alone, as indicated. Error bars, ± SEM, **P<0.01. C. Immunostaining of pulp tissues from dogs with mild pulpitis using anti-MHC class II IgG on the indicated days after treatment with MMP-3, MMP-3 plus NNGH, NNGH alone or saline alone, as indicated. Scale bar, 100 µm. D. Quantitative analysis of the MHC class II-positive cells on the indicated days after treatment with MMP-3, MMP-3 plus NNGH, NNGH alone or saline alone, as indicated. Error bars, ± SEM, **P<0.01.

The changes in the recruitment of macrophages and antigen-presenting cells in the mild pulpitis model were evaluated by immunohistochemical analyses using CD68 and MHC class II antibodies, respectively. High numbers of CD68- and MHC class II-positive cells were observed in the pulp tissues at 1 day after treatment with MMP-3, MMP-3 plus NNGH, NNGH alone or saline alone (Figure 4). However, the number of CD68-positive cells at 3 days after treatment exhibited a significant 1.5-fold decrease in the MMP-3-treated pulp tissues compared with the pulp tissues treated with MMP-3 plus NNGH, NNGH alone or with saline alone (Figure 4A, B). There were very few CD68-positive cells in the pulp tissues at 7 days after MMP-3 treatment (Figure 4A, B). The number of MHC class II-positive cells at 3 days after treatment was also decreased by approximately 1.7-fold in the MMP-3-treated pulp tissues compared with the pulp tissues treated with MMP-3 plus NNGH, NNGH alone or with saline alone (Figure 4C, D). The number of MHC class II-positive cells at 7 days after MMP-3 treatment was also negligible (Figure 4C, D), similar to what was observed for the CD68-positive cells.

### Decreased IL-6 Expression at 3 Days after MMP-3 Treatment

**Figure 5:**
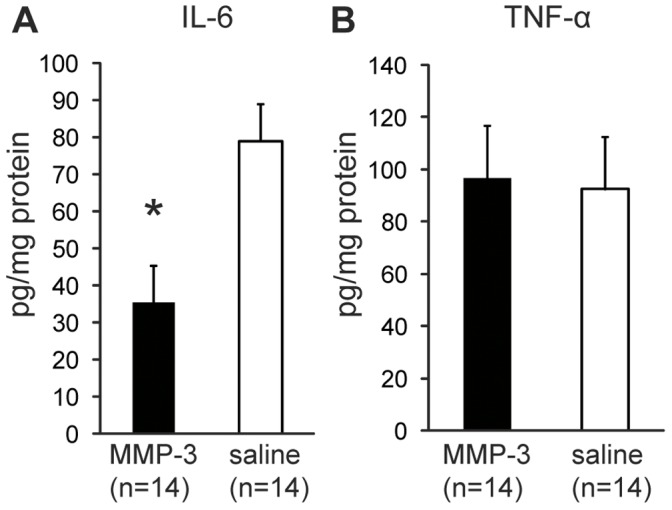
Levels of IL-6 and TNF-α at 3 days after MMP-3 treatment in mild pulpitis model. Homogenates of pulp tissues were prepared at 3 days after MMP-3 or saline treatment as indicated. A. The average concentration (ng/mg protein) of IL-6 is indicated. B. The average concentration (ng/mg protein) of TNF-α is indicated. Error bars, ± SEM, *P<0.05.

As demonstrated by the immunohistochemical analysis of CD68 and MHC class II, the number of inflammatory cells began to decrease 3 days after the MMP-3 treatment. Therefore, we further examined pulp tissues 3 days after treatment by measuring the concentration of the inflammatory cytokines IL-6 and TNF-α. The IL-6 expression levels in the pulp tissues of the MMP-3-treated teeth were significantly reduced (35.52±9.08 pg/mg protein) at 3 days after sealing compared with the saline-treated teeth in the mild pulpitis model (78.95±10.09 pg/mg protein) (mean ± SEM, Figure 5A). In contrast, the levels of TNF-α at 3 days after MMP-3 or saline treatment in the mild model were 96.79±20.23 or 92.40±21.76 pg/mg protein, respectively (mean ± SEM, Figure 5B). There was no significant difference in the levels of TNF-α between the MMP-3- and saline-treated pulp tissues. The pulp tissues in the severe pulpitis model at 3 days after MMP-3 or saline-treated were already necrotic; we could not detect either IL-6 or TNF-α protein (data not shown).

### The Localization of Hyaluronan (HA), Serum-derived HA-associated Protein (SHAP) and Versican in the MMP-3 Treated Mild Pulpitis Tissues

**Figure 6:**
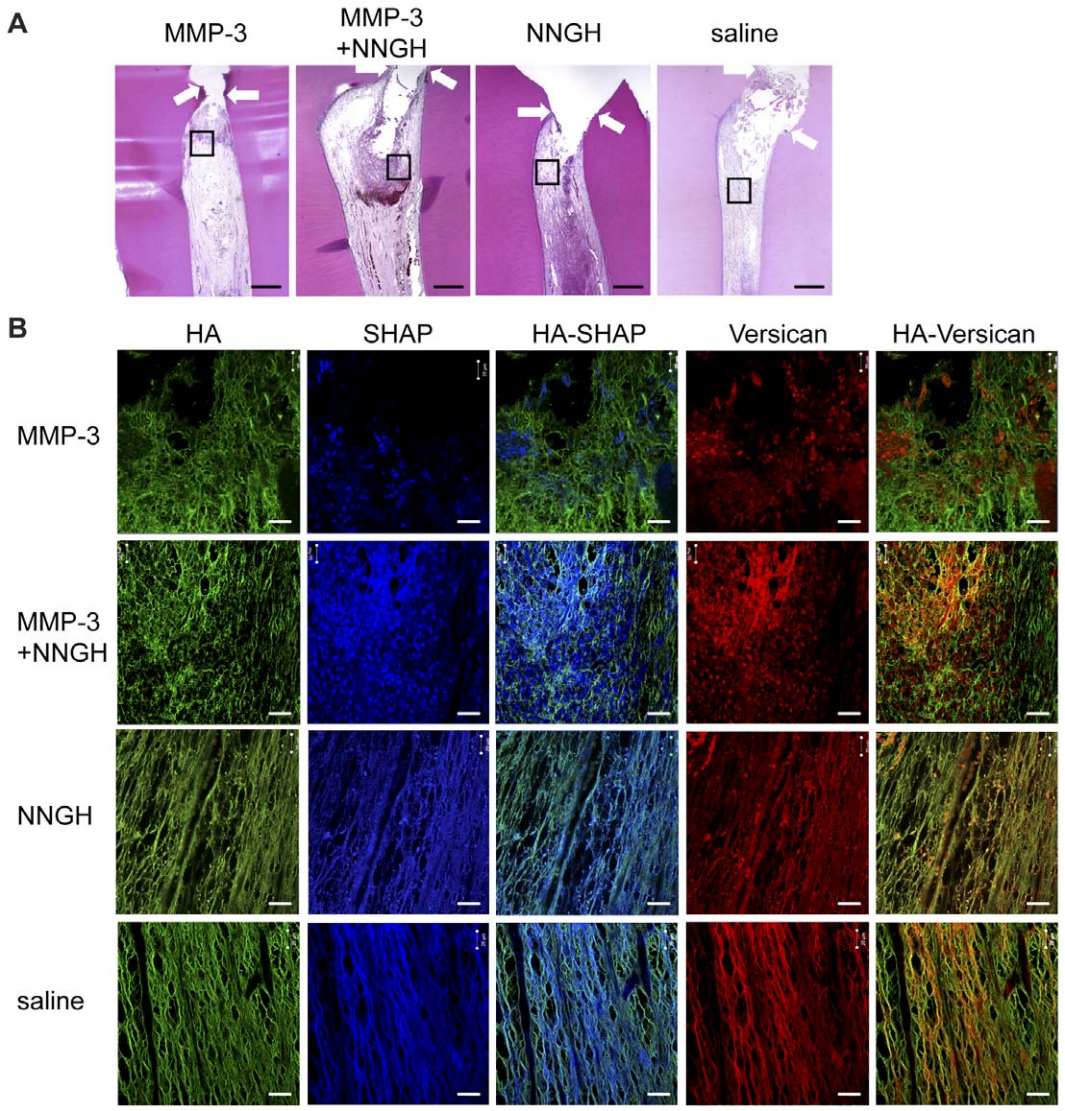
Hyaluronan (HA), SHAP and versican localization in the mild pulpitis model. Immunofluorescence-stained pulp tissues were prepared 3 days after treatment. A. H&E-stained pulp tissues from dogs with mild pulpitis 3 days after treatment with MMP-3, MMP-3 plus NNGH, NNGH alone or saline as indicated, with the indicated area magnified in B. The arrows indicate the imaginary amputated site. Scale bar, 200 µm. B. Pulpitis tissues stained with biotinylated HABP (HA), anti-SHAP (SHAP) and anti-versican as indicated. Scale bar, 20 µm.

As demonstrated by the immunohistochemical analyses and the enzyme-linked immunosorbent assay, the number of inflammatory cells and the expression of IL-6 in MMP-3-treated teeth were significantly decreased 3 days after the MMP-3 treatment. We therefore examined the extracellular matrix changes underneath the amputated site induced by MMP-3 in mild pulpitis tissues 3 days after treatment. Immunofluorescence staining demonstrated that HA co-localized with SHAP and versican underneath the amputated site in pulpitis tissues treated with MMP-3 plus NNGH, NNGH alone or saline. However, in the same region in MMP-3-treated pulpitis tissues, HA did not co-localize with SHAP or versican (Figure 6).

## Discussion

In our previous study, the therapeutic application of MMP-3 for the injured pulp tissues of rat incisors successfully induced tissue regeneration [15]. However, the continuously erupting rodent incisors exhibited significantly different pulp tissue organization compared with mature erupted teeth [16]. The injured pulp tissues of rodent incisors exhibited significantly shorter healing times compared with mature canine teeth [17], and therefore, the injury responses of rat incisors might be quite different to mature teeth. The introduction of bacteria into the pulp of mature teeth can easily cause irreversible pulpitis that impairs the spontaneous healing of the pulp tissue, ultimately resulting in the necrosis and death of the pulp tissue. Therefore, we have extended our investigation using a canine mature erupted premolar model to determine the role of MMP-3 in the inflammation and damage of dental pulp tissues. In addition, the dental, periodontal, and neural reactions to chronic pulpitis and pulpal necrosis have previously been investigated in a model of infected pulpitis triggered by the exposure of pulp tissues [18]. However, there have been no studies that have determined or modeled the sequential stages of irreversible pulpitis. In this study, we successfully established models of mild and severe irreversible pulpitis by exposing the injured pulp of canine mature premolars for 24 and 72 hours, respectively. These models were then used for the evaluation of anti-inflammatory agents.

In the mild irreversible pulpitis model, the regeneration of pulp tissue with its associated vasculature and nerves was observed until 14 days after sealing with MMP-3, followed by extracellular matrix formation in the regenerated pulp tissues until day 28, consistent with our previous report [15]. We followed the histological progression of the mild pulpitis model for 7 days after treatment to further assess the function of MMP-3 in the inflamed pulp tissues. Interestingly, the number of inflammatory cells present 1 day after the treatment was significantly reduced 3 days after the treatment. The inflammatory cells could no longer be detected 7 days after the treatment. Immunohistochemical analysis revealed that the number of CD68-positive macrophages and MHC class II-positive cells was also reduced in the MMP-3-treated pulp tissues. The catalytic cleavage of the MMP-3 substrates appeared to be critical, as demonstrated by the fact that an MMP inhibitor, NNGH, abolished these anti-inflammatory effects. In our previous study, MMP-3 was found to be up-regulated in the rat incisor model 12 and 24 hours after pulp injury, whereas the expression of MMP-2, MMP-9, MMP-10 and MMP-14 was found to be negligible [15]. These data suggested that MMP-3 has a unique role in pulpitis that the other MMPs do not share. Although the MMP-3 stimulated angiogenesis and accelerated pulp regeneration observed in our mild irreversible pulpitis canine model might be useful in future therapeutic modalities, the precise mechanisms underlying the anti-inflammatory effects of MMP-3 remain unclear. In contrast to the mild pulpitis model, the treatment with MMP-3 could not induce tissue regeneration once the inflammation reached the central pulp in the severe pulpitis model. In the severe pulpitis model, the pulp tissues exhibited necrosis at 3 days even after MMP-3 treatment. Therefore, treatment with MMP-3 only elicited anti-inflammatory effects when the inflammation was limited to the upper regions of the pulp.

During infection and certain pathologic conditions, polarized Th1 and Th2 responses are believed to be critical to the outcome of these conditions [19], [20]. Understanding the status of the Th1 and Th2 responses should therefore provide insight into the pathogenesis of such diseases. We therefore analyzed the expression levels of a Th1 cytokine (TNF-α) and a Th2 cytokine (IL-6) in the MMP-3 treated pulpitis tissues. Notably, MMP-3 selectively inhibited the expression of IL-6 but not of TNF-α. This result suggests that MMP-3 may be specifically involved in the Th2 response and that the Th1 response may be sufficient to protect against mild pulpitis. An immune response with mixed Th1 and Th2 properties is unable to provide protection [21]. Thus, the suppression of the Th2 response by MMP-3 may be critical in the host protection response. Recent studies have also demonstrated that IL-6, in concert with TGF-beta, induces the development of Th17 cells from naïve T cells; in contrast, IL-6 inhibits the TGF-beta-induced Treg differentiation [22]. Overproduction of IL-6 is associated with chronic inflammatory diseases such as multiple sclerosis and rheumatoid arthritis, in which the Th17 cells are thought to be the primary cause of the pathology [22]. Given the critical role of IL-6 in regulating the balance between the Treg and the Th17 cells, controlling IL-6 activities by MMP-3 is a potentially effective approach to treat not only mild irreversible pulpitis but also various chronic inflammatory diseases.

In many inflammatory situations, HA and HA-binding proteins (HABPs) regulate the functions of surrounding inflammatory cells, including cytokine release, cell migration and apoptosis. In the presence of inflammatory stimuli, serum-derived hyaluronan-associated proteins (SHAPs) are recruited to extravascular sites, where they are bound to locally synthesized HA to form SHAP-HA complexes, which play roles in the interaction of the matrices with inflammatory cells. SHAP-HA complexes in inflammatory tissues enhances the HA receptors CD44-mediated leukocyte adhesion by a factor of 20- to 30-fold [14], [23]. Recent findings indicate that CD44 plays a critical role in the accumulation of Th2 cells, but not of Th1 cells [24]. Versican is another HA binding proteins and carrying the SHAP attached to the glycosaminoglycan (GAG) chains [25] and support binding of SHAP to HA [26], [27]. MMP-3 digests versican by separating the hyaluronan-binding region from the GAG-rich region of the molecule [28]. In the present study, immunofluorescence staining demonstrated that HA co-localized with SHAP and versican underneath the amputated site in pulpitis tissues treated with MMP-3 plus NNGH, NNGH alone or saline. However, in the same region in MMP-3-treated pulpitis tissues, HA did not co-localize with SHAP or versican. These data raise the possibility that versican digested by MMP-3 could not support SHAP binding to HA. Therefore, the anti-inflammatory effect of MMP-3 may be in part due to the modification of SHAP-HA complexes through the degradation of versican by MMP-3. On the other hand, immunofluorescence staining also demonstrated lower-intensity SHAP staining in the MMP-3-treated pulpitis tissues. This data is interesting to speculate that SHAPs are unstable after the digestion of versican by MMP-3 and that versican may have a critical role in the stabilization of SHAP-HA complexes. Further studies are required to clarify the interaction between SHAP and versican.

## Materials and Methods

### Experimental Animals and Surgical Procedures

All animal manipulations were performed in accordance with the Guidelines for Animal Experimentation issued by Japanese Association for Laboratory Animal Science (1987) and approved by the Animal Experimental Ethics Committee of the National Center for Geriatrics and Gerontology and Aichi-Gakuin University. Female beagle dogs (8±1 kg) of 9 months of age were purchased from Nosan Corporation (Yokohama, Japan). The dogs were anesthetized by the intravenous administration of pentobarbital sodium (25 mg/kg, Schering-Plough). The crowns of the upper and lower premolars were removed with a diamond point burr (Shofu), and then the pulp tissues were amputated with a no. 1/2 round burr (Shofu) as shown in the schematic diagrams (Figure 1A). The amputated pulp tissues were thoroughly washed with saline. In the mild pulpitis model, the amputated pulp tissues remained exposed for 24 hours after injury for mild infection, treated with saline (administered with saline-soaked Spongel; Astellas Pharma), and sealed with phosphate cement and light-cured composite resin (GC) after treatment with a bonding agent (Kuraray) (Figure 1A). In the severe pulpitis model, the amputated pulp tissues remained exposed for 72 hours for severe infection and sealed as described for the mild pulpitis model. After these infections but prior to sealing, 50 ng of MMP-3 (Millipore) was applied to the amputated pulp using Spongel and sealed. The enzymic activity of MMP-3 was measured by using the fluorogenic synthetic substrate Mca-Arg-Pro-Lys-Pro-Val- Glu-Avl-Trp-Arg-Lys(Dnp)-NH2 [Mca, (7-methoxycoumarin-4- yl)acetyl; Avl, amino valeric acid; Dnp, dinitrophenyl] [29]. Before application, the concentrations of MMP-3 were determined in assays using fluorogenic synthetic substrates by titration of their activities against recombinant tissue inhibitor of metalloproteinases 1 (TIMP-1; concentration determined by amino acid analysis) as previously described [30]. As a control, Spongel was used to apply saline or a synthetic inhibitor of MMP-3 (NNGH, N-isobutyl-N-(4-methoxyphenylsulfonyl)-glycylhydroxamic acid, Biomol) together with MMP-3 (molar ratio: 3∶1). After the treatment, the cavities were sealed with phosphate cement and resin as described above.

### Histological Analyses

For morphological analysis, a total of 78 teeth from 24 dogs were used. The teeth were extracted under anesthesia on days 1, 3, 7, 14 and 24, fixed overnight in 4% paraformaldehyde at 4°C, and decalcified with Kalkitox (WAKO Chemicals) according to the manufacturer’s protocols. The samples were dehydrated using a graded ethanol series and embedded in paraffin wax (Sigma). Next, 5 µm-thick sections were cut and mounted on aminopropyl triethoxysilane-coated slides (Matsunami). The paraffin sections were examined after H&E staining or Masson trichrome staining.

### Immunohistochemistry

Immunohistochemical analysis was performed on MMP-3-treated samples to determine the levels of BS-1 lectin, TuJ1, and GAP43 on day 28 as well as the levels of CD68 and MHC class II in MMP-3-treated, MMP-3 plus NNGH-treated and saline control samples on days 1, 3, and 7. The sections were treated with 0.3% hydrogen peroxide in PBS for 30 min at room temperature. After blocking with 10 mg/ml blocking reagent (PerkinElmer) for 1 hour at room temperature, the samples were incubated with FITC-conjugated BS-1 lectin (Vector Laboratories), anti-TuJ1 (Covance), anti-GAP43 (Chemicon), anti-CD68 (Abcam) or anti-MHC class II (AbD Serotec) for 1 hour at room temperature. As a negative control, non-immune IgG of the same dilution was used in place of the primary antibodies. The sections were rinsed in PBS and incubated with the horseradish peroxidase (HRP)-conjugated secondary antibodies (Vector Laboratories) for 30 min at room temperature. The sections were washed with PBS and then immersed in a diaminobenzidine solution (Vector Laboratories) for 10 min at room temperature to visualize the immunoreactivity. The number of immunoreactive cells was evaluated in 10 fields for every section from 6 teeth of each group at 400x magnification (0.1 mm^2^). Counting immunoreactive cells was performed by using BZ-II Analyzer software (Keyence). The data were presented as the mean ± SEM of the immunoreactive cells. Immunofluorescent staining with biotinylated HABP, anti-SHAP antibodies and anti-versican antibodies was performed as described previously [23], [31], [32].

### Enzyme-linked Immunosorbent Assay (ELISA)

For enzyme-linked immunosorbent assay analysis of inflammatory cytokines, a total of 28 teeth from 7 dogs were used. The samples of pulp tissue homogenates were prepared from 2 premolars extracted from the same dogs at 3 days after treatment with MMP-3 or saline. Seven samples from each treatment group were prepared for analysis. The concentrations of IL-6 and TNF-α were determined using ELISA kits (R&D Systems) according to the manufacturer’s protocols.

### Data Analysis

All of the data are presented as the mean values ± SEM. The differences between groups were tested for statistical significance using the two-tailed Mann-Whitney *U* test. P-values <0.05 were considered statistically significant.
